# Disease burden of tuberculosis in China from 1990 to 2021 and its prediction to 2036

**DOI:** 10.3389/fpubh.2024.1506266

**Published:** 2025-01-07

**Authors:** Rong Sun, Liang Wang, Hongfang Xia

**Affiliations:** ^1^Clinical Laboratory, China University of Geosciences Wuhan Hospital, Wuhan, China; ^2^Department of Public Health, Wuhan Sports University Hospital, Wuhan, China; ^3^Department of Public Health, China University of Geosciences Wuhan Hospital, Wuhan, China

**Keywords:** tuberculosis, joinpoint analysis, age-period-cohort analysis, BAPC model, disease burden, GBD

## Abstract

**Background:**

Tuberculosis (TB) is one of the oldest infectious diseases and continues to be a major killer of human beings. This paper was designed to provide insights into the disease burden of TB.

**Methods:**

The data was retrieved and downloaded from the latest GBD database. Joinpoint regression was done for the temporal trend analysis. The age-period-cohort model was introduced to get further insights into the independent effects of age, period, and cohort. The BAPC model was utilized to predict ASIR and ASMR from 2022 to 2036.

**Results:**

From 1990 to 2021, the ASPR dropped from 31,446 (95% UI: 27,902 to 35,142) to 30,557 (95% UI: 27,693 to 33,531) per 100,000 people, and ASDALYR dropped from 719 (95% UI: 611, 837) to 76 (95% UI: 63, 94) per 100,000 people with an AAPC of −7.009 (95% CI: −7.219, −6.799). ASIR and ASMR decreased from 109 (95% UI: 95, 125) to 36 (95% UI: 33, 40) and from 20 (95% UI: 17, 24) to 2 (95% UI: 2, 3) per 100,000 people, respectively. Men had a higher TB burden than women. The age-period-cohort analysis showed the age effect represented significant fluctuations with a valley at age 5 for incidence rate, and a similar but relatively simple pattern for death rate. Period effect and cohort effect showed both incidence and mortality rates significantly decreased with advancing time points and more recent birth cohorts. At the current decline rate, the ASIR and ASMR would be 26.12 (95%CI: 15.75, 36.48) per 100,000 people and 1.13 (95%CI: 0.45, 1.81) per 100,000 people in 2030, respectively. And the ASIR would be 21.96 (95%CI: 6.14, 37.79) per 100,000 people in 2035.

**Conclusion:**

TB burden in China has decreased significantly overall in the past years. However, it is still hard to achieve the national goal of “End TB” by 2035, which means more effective strategies for TB prevention and control are urgently needed. Effective strategies aimed at men should include increasing awareness of tuberculosis among both the general population and healthcare workers, promoting smoking cessation and alcohol reduction, enhancing disease screening and treatment access, and providing psychological support and care.

## Introduction

Tuberculosis (TB) is an airborne infectious disease caused by the *Mycobacterium tuberculosis* complex. While it primarily affects the lungs, TB can also impact nearly any part of the body (1). The disease spreads mainly through the air, particularly when individuals with active TB of the lungs or throat talk loudly, sing, cough, sneeze, or spit. When they do, the bacteria are released into the air, where they can remain for several hours, depending on the environment. People who inhale air containing these TB germs can become infected; this is known as latent TB infection. This transmission can occur among family members or coworkers of the infected individual (2). The epidemic state of TB decreased substantially in China in the past few decades (3, 4). However, as described in the “Global Tuberculosis Report 2023,” an estimated 10.6 million people fell ill with TB in 2021, an increase of 4.5% from 2020, and 1.6 million people died from TB (including 187,000 among human immunodeficiency virus positive people) (5), which means a deviation from the Global Plan to Stop TB by 2035. China, as one of the 30 high TB burden countries, has made remarkable progress in controlling the disease through a combination of robust policies, improved health services, and increased public awareness. However, it was still among the eight countries which accounted for two thirds of the global TB burden in 2022, suggesting TB remains a significant public health concern for China, particularly in rural and impoverished areas (6).

Understanding the changing burden of TB and its underlying drivers is crucial for designing effective strategies to combat this disease. The incidence rate refers to the number of new cases of a disease or condition that occur in a specified population over a specific period of time. It is useful for identifying trends in disease occurrence and for comparing disease rates across different populations. On the other hand, the prevalence rate measures the total number of existing cases of a disease or condition within a specified population at a certain point in time. This rate helps assess the extent of a disease or condition in the population. The mortality rate indicates the number of deaths from a specific cause within a defined population over a specific period of time. This statistic is used to measure the frequency of deaths attributable to that cause. Another important measure is the disability-adjusted life years rate (DALYR), which combines both mortality and morbidity into a single metric. This measure quantifies the number of years lost due to illness, disability, or premature death, providing an overall assessment of the burden of disease, including both fatal and non-fatal outcomes. Furthermore, age-standardized rates—including age-standardized incidence rate (ASIR), age-standardized prevalence rate (ASPR), age-standardized mortality rate (ASMR), and age-standardized disability-adjusted life years rate (ASDALYR)—adjust for age structure. These rates enable comparisons across different populations or time points by accounting for the impact of age. Inadequate healthcare resources, socioeconomic disparities, and changes in the spectrum of infectious diseases, the ongoing pandemic of corona virus disease 2019 (COVID-19) for example, has further complicated TB control efforts, disrupting health services and exacerbating the burden of TB globally (7, 8). Moreover, demographic shifts, including aging populations and increased urbanization, are also influencing the epidemiology of TB. Therefore, continuous in-depth analyses of the TB burden in China, along with its global context, are essential for developing targeted interventions and predicting future trends. So, the Global Burden of Disease Database (GBD) 2021 was mined to obtain the latest insights into the current situation and future trends of TB burden.

This paper starts with a review of the global and Chinese tuberculosis (TB) burden from 1990 to 2021, comparing this data to existing studies to contribute to the ongoing discussion on TB control and prevention. We then explore the interactive and independent effects of age, period, and cohort on TB disease burden trends. Following this analysis, we present a predictive analysis of TB trends using Bayesian age-period-cohort (BAPC) models. This aims to provide insights into the future burden of TB and its potential implications for public health.

## Materials and methods

### Data sources

The GBD database compiled and analyzed a vast array of data on the health status and trends of populations worldwide, providing valuable insights into the burden of diseases, injuries, and the underlying risk factors that contribute to ill health across countries, regions, and age groups. Updated periodically, the latest GBD database (i.e., GBD2021) leverages lots of advanced statistical modeling to estimate disease burden for 371 diseases and injuries, as well as 88 risk factors, spanning from infectious diseases to non-communicable conditions like cardiovascular disease, cancer, and mental health disorders in 204 countries and territories and 811 sub-national locations from 1990 to 2021. A thorough introduction to GBD2021 has been reported in relevant high-quality literature (9–11). The TB data, including the estimates and 95% uncertainty intervals (UIs) of the disease burden was downloaded from the GBD2021 database, which was available at https://vizhub.healthdata.org/gbd-results/ (12).The main variables this research analyzed are as follows:

- Age-Standardized Incidence Rate (ASIR):
  - Adjusts for age differences in the occurrence of diseases.
  - Provides a comparable measure of new cases across different populations.
  - Crucial for monitoring disease trends and evaluating the effectiveness of interventions.
- Age-Standardized Mortality Rate (ASMR):
  - Adjusts for age when measuring deaths from specific causes.
  - Reflects the impact of diseases on mortality patterns.
  - Important for policy formulation and resource allocation.
- Age-Standardized Prevalence Rate (ASPR):
  - Adjusts for age in measuring the proportion of a population affected by a disease.
  - Indicates the burden of disease within a population.
  - Useful for identifying high-risk groups and guiding interventions.
- Age-Standardized Disability-Adjusted Life Years Rate (ASDALYR):
  - Combines years lost due to premature mortality and disability.
  - Adjusts for age and severity of disease.
  - Provides a comprehensive measure of overall disease burden.

Due to public accessibility to the data, ethics approval and informed consent were not required for this study. Meanwhile, the GBD open-source dataset has several limitations that stem from the characteristics of the data and the methods used for its collection and processing. Firstly, while the GBD dataset is comprehensive, it may not fully capture the nuances and variations in disease burden across different geographical regions and populations. Since the data is aggregated at a global level, it lacks detailed information for various regions in China, making it difficult to analyze the health burden of TB by region and ethnic groups. Secondly, the methodologies used to estimate disease burden within the GBD framework rely on a series of assumptions and models, which can introduce biases and uncertainties. These assumptions may not always align with specific contextual realities, potentially leading to inaccuracies in the estimates. Therefore, caution is advised when drawing conclusions from secondary analyses based on GBD data. Finally, the timeliness and currency of the GBD dataset can also be a limitation. The data might not always reflect the most recent trends in disease burden due to delays in collection, processing, and publication.

### Statistical analysis

#### Descriptive statistical analysis

This study conducted descriptive analysis and visualization of the temporal trend of TB epidemiological characteristics, such as age-standardized incidence rate (ASIR), age-standardized prevalence rate (ASPR), age-standardized mortality rate (ASMR), and age-standardized disability-adjusted life years rate (ASDALYR), focusing on the differences between China and the World, as well as the differences among different genders and ages in China. All analyses and visualizations were performed using R software (version 4.2.1).

#### Joinpoint regression analysis

To more precisely reveal the temporal trends in ASIR, ASPR, ASMR, and ASDALYR of TB from 1990 to 2021, the Joinpoint 5.1.0.0 software which was available on the website of the National Cancer Institute (accessed on 10 April 2024),^1^ was employed to conduct segmented regression analysis (13). This method can identify significant joinpoints, segmenting the overall long-term trends into distinct subsegments (14). In this study, we also calculated and assessed the annual percentage change (APC) with their 95% confidence interval (CI) and the significance of the trends for each subsegment. Additionally, the average annual percentage change (AAPC) was calculated to summarize the overall trends. If the lower boundary of APC/AAPC 95%CI is found to be greater than 0, signified an uptrend within the respective subsegment; conversely, the upper boundary of its 95%CI falling below 0 demonstrated a downtrend. A *p-*value of less than 0.05 was considered statistically significant.

#### Age-period-cohort analysis

To further disentangle and quantify the independent and interactive effects of age, period, and cohort on the temporal trends in ASIR and ASMR of TB from 1990 to 2021, this study adopted the Bayesian age-period-cohort (BAPC) models as a robust methodological framework, which was a statistical tool rooted in Bayesian inference and had gained widespread recognition among epidemiologists, demographers and public health researchers (15). The BAPC model is based on age-period-cohort analysis, which describes the change trend of diseases according to the impact of age, period and cohort on morbidity or mortality. Utilizing Bayesian inference techniques, the model estimates the posterior distribution by combining prior information about unknown parameters with sample data, allowing for the inference of unknown parameters based on this posterior distribution.

However, there is collinearity among the three factors in the age-period-cohort model, which makes parameter estimation difficult. In this study, the Intrinsic Estimator (IE) method was utilized to address the complete collinearity relationship between age, period, and cohort variables (16, 17). Through the BAPC model, we can estimate the effect coefficients of three factors and convert them into relative risk (RR). By plotting the changes in RR over time for each factor, we can intuitively examine the underlying patterns and trends related to age, period, or cohort. This approach helps us discover the magnitude and direction of each factor’s impact on the outcome. To meet the requirements of age-period-cohort analysis, the data were organized into successive 5-year age groups (i.e., 0–4, 5–9, …, 95–99), consecutive 5-year periods from 1992 to 2021 (i.e., 1992–1996, 1997–2001, …, 2017–2021) and corresponding consecutive 5-year birth cohorts (i.e.,1897–1901, 1902–1906, …, 2017–2021). And then, we, respectively, computed the relative risk (RR) for age, period, and cohort. Stata software (version 15.0) was used for age-period-cohort analysis and R software (version 4.2.1) was used for visualizations.

#### Forecasting ASIR and ASMR beyond 2021

Furthermore, the BAPC model’s predictive power is widely accepted. By leveraging historical data and accounting for uncertainty, the model can generate forecasts of future trends in health outcomes, including mortality or incidence (18–20). In this study, we applied the BAPC model to forecast the ASIR and ASMR of TB in China from 2022 to 2036. The standard population data used in the model was sourced from World Standard (WHO 2000–2025), which was available^2^ (21). All analyses and visualizations were performed by R software (version 4.2.1).

## Results

### Disease burden of TB and its temporal trend

**Figure 1:**
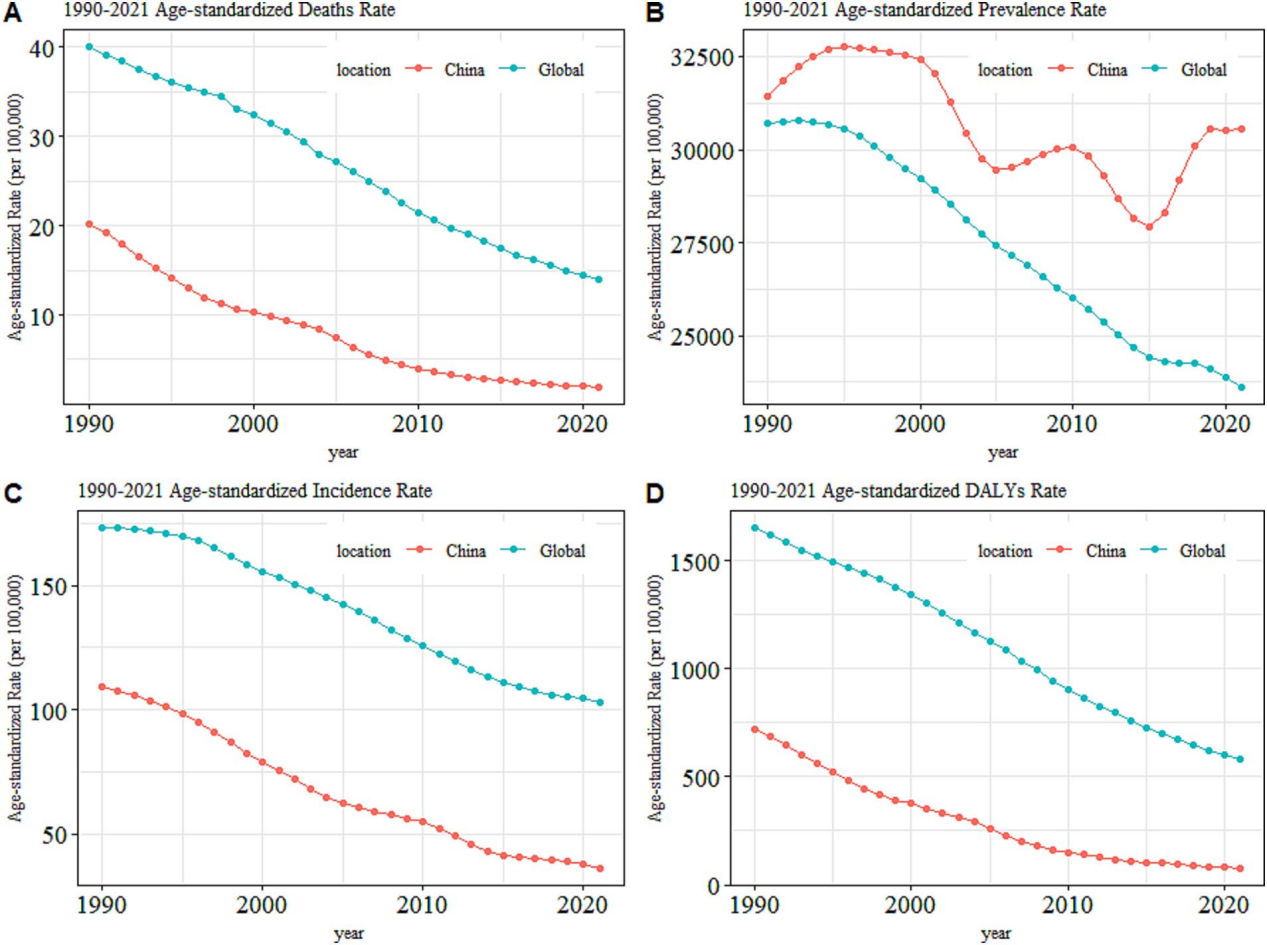
The overall trends in the age-standardized rates for TB in China versus that at the global level, **(A)** Mortality, **(B)** Prevalence, **(C)** Incidence, and **(D)** DALYs.

**Figure 2:**
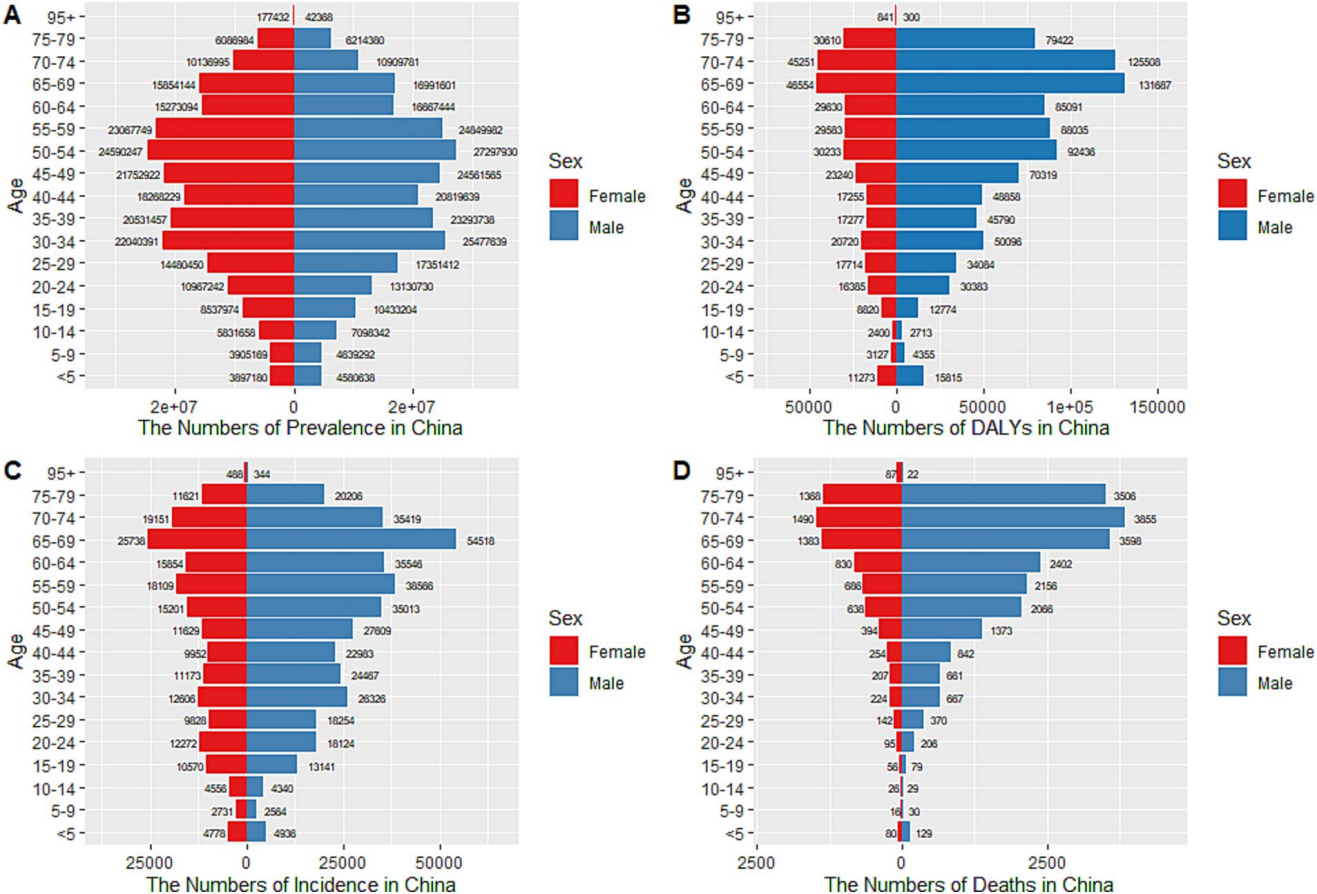
Age-specific numbers of prevalence, DALYs, incidence, and deaths for TB in China, 2021. **(A)** Age-specific prevalence number, **(B)** Age-specific DALYs number, **(C)** Age-specific incidence number, **(D)** Age-specific death number.

**Figure 3:**
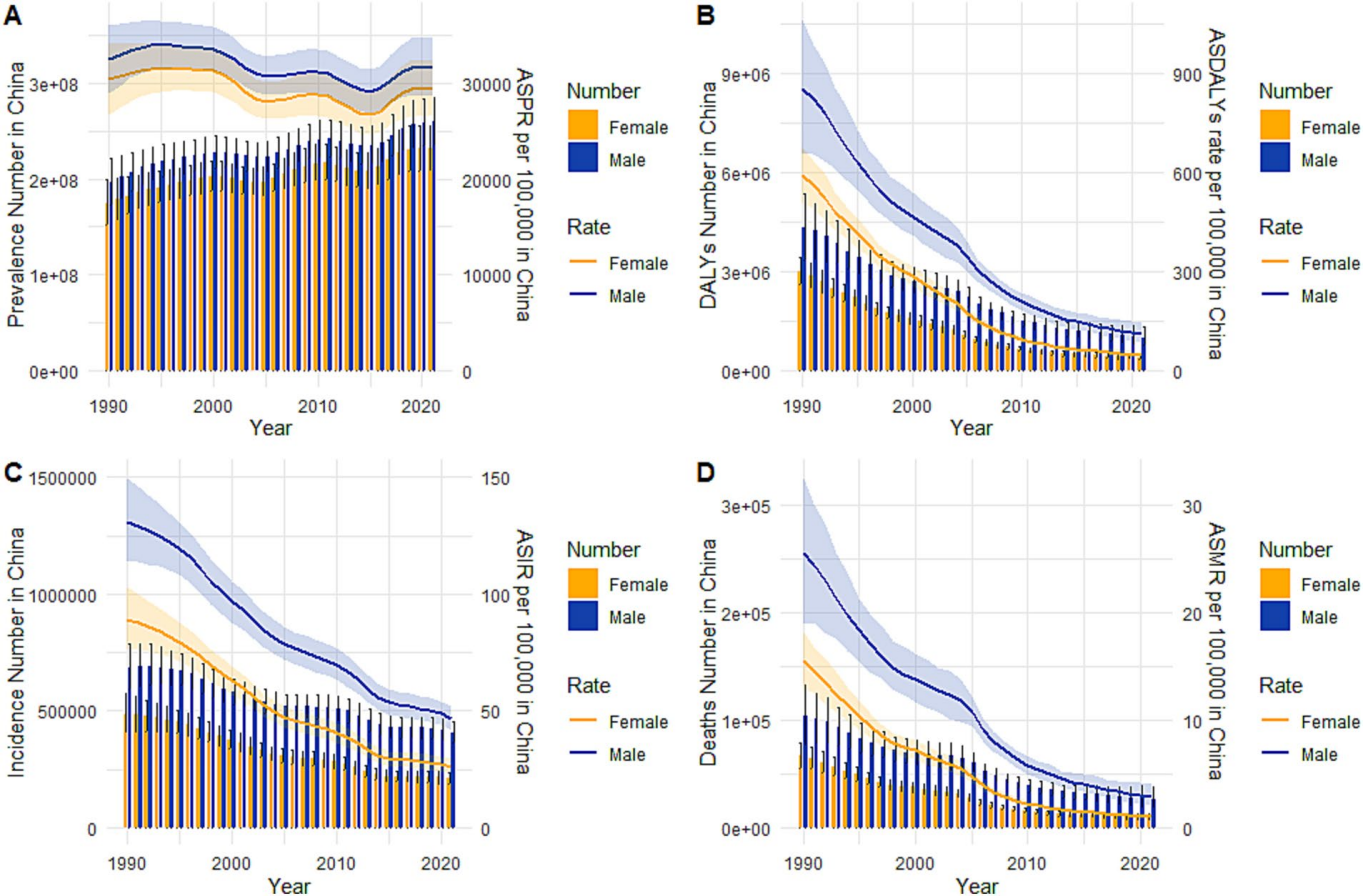
Trends in the all-age cases and age-standardized incidence, prevalence, mortality and DALYs rates of TB by sex from 1990 to 2021. **(A)** Prevalence number and rate, **(B)** DALYs number and rate, **(C)** Incidence number and rate, **(D)** Mortality number and rate.

In China, TB led to 617,726 (95% UI: 549,548 to 688,348) new cases and 37,332 (95% UI: 29,309 to 49,368) deaths in 2021 as per the GBD2021 data. Meanwhile, the ASIR, ASMR, and ASDALYR for TB in 2021 were 36 (95% UI: 33, 40) per 100,000 people, 2 (95% UI: 2, 3) per 100,000 people, and 76 (95% UI: 63, 94) per 100,000 people respectively, reflecting AAPCs of −3.491 (95% CI: −3.609, −3.372), −7.424 (95% CI: −7.777, −7.069), and − 7.009 (95% CI: −7.219, −6.799) compared to 1990 (Table 1). The ASPR for TB in 2021 was 30,557 (95% UI: 27,693 to 33,531) per 100,000 people with an AAPC of −0.152 (95% CI: −0.366, 0.063) compared to 1990, indicating the decline in ASPR was not statistically significant (*p* = 0.167). At the global level, the ASIR, ASPR, ASMR, and ASDALYR for TB in 2021 were 103 (95% UI: 92, 115) per 100,000 people, 23,614 (95% UI: 21,451 to 26,020) per 100,000 people, 14 (95% UI: 13, 16) per 100,000 people, and 580 (95% UI: 522, 650) per 100,000 people respectively, reflecting AAPCs of −1.655 (95% CI: −1.740, −1.570), −0.850 (95% CI: −0.912, −0.787), −3.340 (95% CI: −3.491, −3.189), and − 3.316 (95% CI, −3.426, −3.206) compared to 1990. Compared with the global disease burden of TB, the ASIR, ASMR, and ASDALYR in China were lower than the global level, and the decline in these metrics in China was larger than that observed worldwide. However, the ASPR in China was higher than the global level (Figure 1). Figure 2 shows the sex-specific prevalence (Figure 2A), disability-adjusted life years (Figure 2B), incidence (Figure 2C), and mortality (Figure 2D) numbers of TB in China for different age groups in 2021. TB was more prevalent in people between the age of 15 and 74. A similar tendency was observed in the incidence and disability-adjusted life years (DALYs), which showed a decrease at the age of 5 and 70, respectively, and an overall increase between the age of 9 and 69 with slight up and down at some ages. The highest incidence peaks occurred in the age group of 65–69. Moreover, the incidence and DALYs of the population aged 69 and above still maintain a high level. In terms of death, numbers stayed at a low level before the age of 19, and increased to higher levels between the age of 20 and 79. After the age of 95, the mortality number decreased dramatically. Men had higher incidence, mortality, and DALYs than women while the difference between men and women was slight for prevalence, which could also be drawn from Figure 3.

**Table 1:** The disease burden presented with cases and ASR for tuberculosis by sex at global level and in China, AAPC from 1990 to 2021.

Measure	Sex	1990		2021		1990–2021 AAPC (95% CI)	*p* value for AAPC
		All-ages cases n (95% UI)	ASR per 100,000 people (95% UI)	All-ages cases n (95% UI)	ASR per 100,000 people (95% UI)		
**China**							
Incidence	Male	683,686 (593,332,789,780)	131 (114,150)	406,433 (360,732,452,642)	47 (42,52)	−3.270 (−3.350, −3.190)	<0.001
	Female	484,122 (407,126,574,804)	89 (77,103)	211,293 (186,374,236,357)	26 (23,29)	−3.845 (−4.094, −3.594)	<0.001
	Total	1,167,808 (1,001,441,1,359,621)	109 (95,125)	617,726 (549,548,688,348)	36 (33,40)	−3.491 (−3.609, −3.372)	<0.001
Prevalence	Male	196,212,727 (172,953,351,221,591,670)	32,457 (28,905,36,013)	259,399,170 (235,884,026,284,569,387)	31,679 (28,789,34,672)	−0.025 (−0.227, 0.176)	0.805
	Female	173,567,058 (151,658,158,198,844,278)	30,407 (26,810,34,280)	232,149,210 (210,320,898,256,071,816)	29,399 (26,568,32,460)	−0.175 (−0.362, 0.012)	0.067
	Total	369,779,785 (324,342,084,420,948,319)	31,446 (27,902,35,142)	491,548,380 (445,855,387,539,371,580)	30,557 (27,693,33,531)	−0.152 (−0.366, 0.063)	0.167
Deaths	Male	103,742 (77,568,132,622)	25 (19,33)	26,981 (19,576,38,551)	3 (2,4)	−6.903 (−7.122, −6.684)	<0.001
	Female	67,349 (56,026,79,053)	16 (13,18)	10,351 (7,976,13,860)	1 (1,1)	−8.457 (−8.743, −8.171)	<0.001
	Total	171,091 (141,634,204,908)	20 (17,24)	37,332 (29,309,49,368)	2 (2,3)	−7.424 (−7.777, −7.069)	<0.001
DALYs	Male	4,325,971 (3,342,350,5,378,669)	855 (661,1,064)	991,182 (767,265,1,335,566)	109 (85,145)	−6.467 (−6.687, −6.247)	<0.001
	Female	2,999,759 (2,585,588,3,434,183)	593 (510,678)	384,328 (308,366,483,222)	45 (36,56)	−8.058 (−8.429, −7.685)	<0.001
	Total	7,325,730 (6,221,452,8,528,411)	719 (611,837)	1,375,510 (1,120,820,1,723,072)	76 (63,94)	−7.009 (−7.219, −6.799)	<0.001
**Global**							
Incidence	Male	4,467,133 (3,942,667,5,108,724)	190 (168,217)	4,684,699 (4,183,366,5,243,434)	115 (104,129)	−1.601 (−1.66, −1.542)	<0.001
	Female	4,131,388 (3,588,419,4,748,723)	160 (140,184)	3,722,434 (3,313,147,4,158,319)	92 (81,103)	−1.760 (−1.837, −1.683)	<0.001
	Total	8,598,520 (7,528,228,9,854,794)	173 (153,199)	8,407,133 (7,519,793,9,393,767)	103 (92,115)	−1.655 (−1.740, −1.570)	<0.001
Prevalence	Male	823,515,218 (738,681,069,911,950,864)	31,391 (28,354,34,503)	987,404,665 (897,029,073,1,085,486,647)	24,386 (22,177,26,832)	−0.812 (−0.881, −0.744)	<0.001
	Female	781,849,757 (701,624,622,865,470,600)	30,032 (27,123,33,116)	924,412,120 (837,484,487,1,015,739,987)	22,848 (20,741,25,214)	−0.884 (−0.961, −0.806)	<0.001
	Total	1,605,364,975 (1,440,332,859,1,777,421,464)	30,697 (27,716,33,777)	1,911,816,784 (1,733,433,144,2,100,447,765)	23,614 (21,451,26,020)	−0.850 (−0.912, −0.787)	<0.001
Death	Male	1,051,722 (810,021,1,227,159)	52 (40,61)	725,385 (646,282,868,622)	18 (16,22)	−3.322 (−3.495, −3.148)	<0.001
	Female	727,147 (660,765,815,158)	30 (28,34)	437,411 (396,771,482,605)	10 (9,11)	−3.456 (−3.6, −3.311)	<0.001
	Total	1,778,869 (1,532,822,1,980,801)	40 (34,45)	1,162,796 (1,050,008,1,313,985)	14 (13,16)	−3.34 0(−3.491, −3.189)	<0.001
DALYs	Male	45,547,389 (35,529,337,53,089,779)	1913 (1,491,2,220)	28,536,132 (25,244,156,33,765,448)	705 (623,834)	−3.158 (−3.278, −3.037)	<0.001
	Female	37,132,385 (33,730,888,41,775,979)	1,424 (1,297,1,598)	18,441,330 (16,705,778,20,317,945)	463 (417,513)	−3.557 (−3.684, −3.431)	<0.001
	Total	82,679,773 (73,004,989,91,407,746)	1,651 (1,458,1825)	46,977,463 (42,482,994,52,463,556)	580 (522,650)	−3.316 (−3.426, −3.206)	<0.001

UI, uncertainty interval; ASR, age standardized rate; AAPC, average annual percentage change; CI, confidence interval; DALYs, disability-adjusted life years.

Figure 3 depicted the trends in the sex-specific all-age numbers and age-standardized rates of TB incidence, prevalence, mortality, and DALYs in China from 1990 to 2021. The sex-specific ASDALYR, ASIR, and ASMR for TB declined gradually (Figures 3B–D) while the ASPR fluctuated by year for both males and females (Figure 3A).

### Joinpoint regression analysis

**Figure 4:**
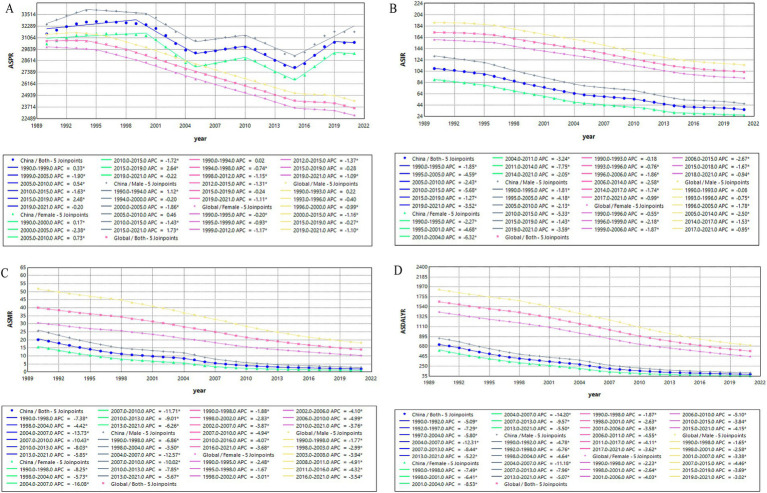
Temporal trends in age-standardized rates of TB in China versus global rates based on the joinpoint regression analysis. **(A)** ASPR, **(B)** ASIR, **(C)** ASMR, **(D)** ASDALYR.

Joinpoint regression analysis of the age-standardized rates for TB in China and globally from 1990 to 2021 were shown in Figure 4. As shown in Figure 4A, compared with the global trend of ASPR, which gradually decreased since 1994, the ASPR in China undulated significantly, specifically decreased from 1999 to 2005 (APC = −1.90), increased in the period 2005–2010 (APC = 0.54), followed a decrease from 2010 to 2015 (APC = −1.63) and then again continuously increased till 2021 with an APC_2015-2019_ = 2.48, despite a non-significant fall in all cases and females (Figure 4A). Different from the trend of ASPR, the ASIR of TB was dropped year after year, with two remarkable drops in the period of 1995–2005 (APC = −4.59) and 2010–2015 (APC = −5.68; Figure 4B). The ASMR significantly decreased from 2004 to 2013 in both of males (APC_2004–2007_ = −12.57, APC_2007-2010_ = −10.02, APC_2010-2013_ = −7.85) and females (APC_2004–2007_ = −16.08, APC_2007-2010_ = −11.71, APC_2010-2013_ = −9.01; Figure 4C). The ASDALYR (Figure 4D) was on a similar downward trend with the ASMR, which also had remarkable declines between 2004 and 2013 (APC_2004–2007_ = −12.31, APC_2007-2013_ = −8.44).

### The effects of age, period, and cohort on incidence/mortality of TB calculated by age-period-cohort models

**Figure 5:**
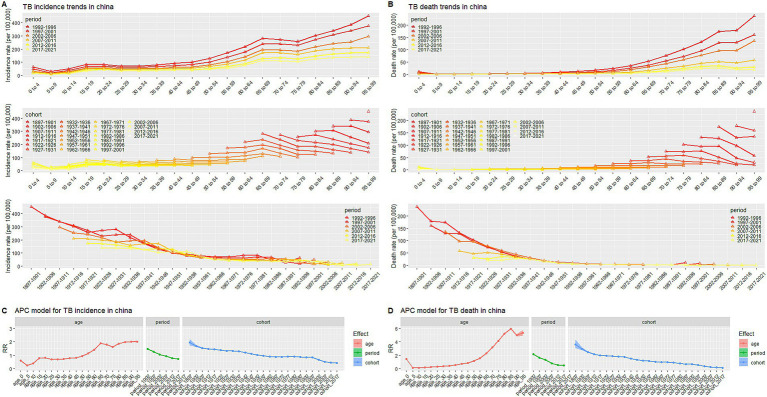
Trends of age-based, period-based and cohort-based variation of TB incidence/mortality rate in China. **(A)** Incidence trends, **(B)** Mortality trends, **(C)** APC model for incidence, **(D)** APC model for mortality. APC model: Age-period-cohort model.

Figure 5 illustrated the age-period-cohort effect on TB incidence and mortality rate. For the overall trend, the TB incidence rate grew up with age, and peaked at the age of 95–99 year-old, which experienced three drops at ages 0–4, 15–34, and 65–74 year-old, respectively. After controlling the effects of period and cohort, it decreased under 5 years old and then increased to the peak. The onset risk of TB at age of 90 (RR = 2.014, 95% CI: 1.980, 2.049) was 8.121 times of that in age 5 (RR = 0.248, 95% CI: 0.246, 0.251). The increase of RR values was initially rapid at the age of 5–15, then slower at the age of 15–95, among which there were two drops at the age of 20–25 and 65–75. The period-based trend of TB incidence gradually decreased with the progress of the times, with the RR value decreased from 1.479 (95% CI: 1.474, 1.483) in period_1992-1996_ to 0.709 (95% CI: 0.707, 0.712) in period_2017-2021_. The birth cohort based trend showed the onset risk was significantly higher in the early birth cohort (RR _cohort 1897–1901_ = 1.945, 95% CI: 1.725, 2.194) and decreased in the recent cohorts (RR_cohort 2017–2021_ = 0.434, 95% CI: 0.426, 0.442) with the maximum reduction in the cohort_1997-2012_. The trend of TB mortality rate was similar to that of incidence rate but with a smaller amplitude, as so as the corrected age effect, period effect, and cohort effect. After controlling for the effects of period and birth cohort, the mortality rate declined with age until 10 years old and then increased, peaking at ages 85–89. However, the age-based incidence and mortality both had an obvious turning point at age 5, the RR were 0.248 (95% CI: 0.246, 0.251) and 0.152 (95% CI: 0.147, 0.158) for incidence and mortality, respectively. The additional effect values of age, period, and cohort were summarized in Supplementary Table S1.

### Prediction of TB incidence and mortality rate from 2022 to 2036 in China

**Figure 6:**
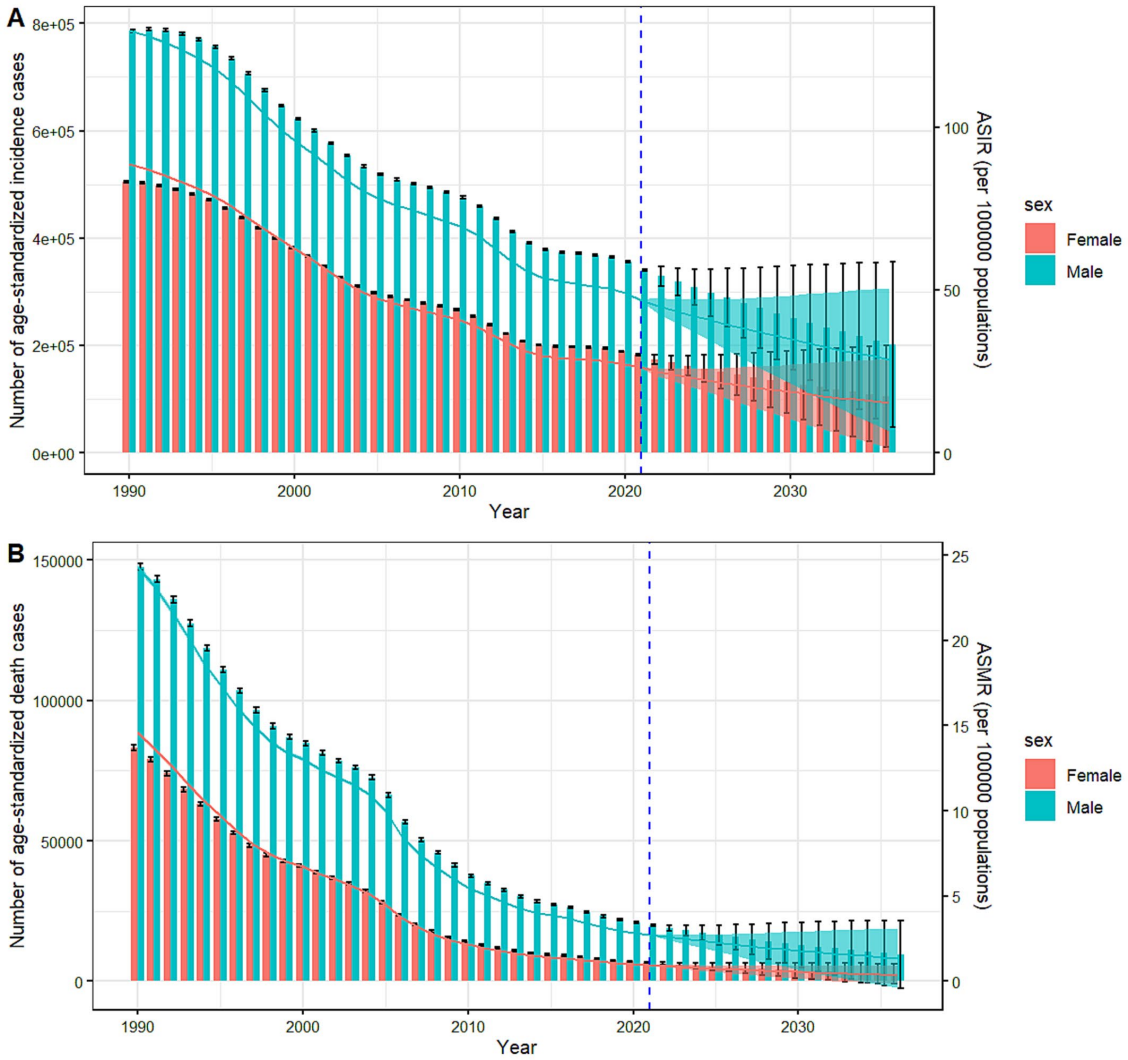
Predicted trends of TB incidence and mortality rate in China from 2022 to 2036. Column and lines before the dotted line represent the true trend of TB incidence/death number and rate respectively; Column and lines after the dotted line represent the predicted trend and its 95% CI. **(A)** Incidence, **(B)** Mortality.

To learn about the trends of TB incidence and mortality after 2021, the BAPC models were used to predict the ASR from 2022 to 2036, the results were summarized in Supplementary Table S2. According to the prediction, the ASIR and ASMR of TB would continue to decline. The ASIR and ASMR would be 26.12 (95% CI: 15.75, 36.48) per 100,000 people and 1.13 (95% CI:0.45, 1.81) per 100,000 people in 2030, respectively. And the ASIR would be 21.96 (95% CI: 6.14, 37.79) per 100,000 people in 2035. Regarding gender stratification, the ASIR in males and females would decrease annually after 2021, from 46.80 (95% CI: 46.65, 46.96) per 100,000 people in 2021 to 29.66 (95% CI: 9.08, 50.22) per 100,000 people in 2035 for males, and from 26.25 (95% CI: 26.13, 26.37) per 100,000 people to 15.83 (95% CI: 2.98, 28.68) per 100,000 people for females, and the ASIR would be 21.96 (95% CI: 6.14, 37.79) per 100,000 people in the year of 2035 for total population (Figure 6A). The predicted mortality rate would also keep going down over the next two decades (Figure 6B), the males’ ASMR was expected to drop from 2.73 (95% CI: 2.70, 2.77) per 100,000 people in 2021 to 1.43 (95% CI: 0.00, 3.07) per 100,000 people in 2035, while the female’ ASMR would go down from 0.96 (95% CI: 0.94, 0.98) per 100,000 people to 0.41 (95% CI: 0.10, 0.91) per 100,000 people. These results indicated that the disease burden of TB in China will gradually decrease in both males and females in terms of ASIR and ASMR.

## Discussion

This study examined the trends of TB burden in China from 1990 to 2021 using the joinpoint analysis combined with the age-period-cohort models and estimated the future trends of TB to 2036 via the BAPC models. The findings indicated an overall decline in TB burden in China as well as the global situation from 1990 to 2021 in terms of ASIR, ASMR, ASPR, and ASDALYR, indicating significant effectiveness in TB control works. However, it is worth noting that since 2010, the downward trends of the TB burden in China have slowed down significantly, particularly in incidence and mortality rates. One potential cause may be comorbidity with diabetes or HIV; the increase in diabetes cases and HIV infection has been identified as significant factors contributing to the sustained TB burden. Patients with diabetes or HIV/AIDS have higher susceptibility to TB (22). Another potential cause may be the economic environment. Since 2010, China’s economic growth has slowed down, partially due to external and cyclical factors, which may have impacted healthcare access and resource allocation for TB control. It is well worth strengthening diabetes management and anti-HIV/AIDS and increasing investment in TB control programs.

Different from the steady decline of ASIR, ASMR, and ASDALYR, the prevalence of TB in China fluctuated significantly during the period analyzed. Through joinpoint regression analysis, it was found that the prevalence of TB in China had experienced several rises and declines from 1990 to 2021. Notably, the significant decline observed during the period from 1999 to 2005 may be attributed to the widespread implementation of the Directly Observed Treatment, Short-course (DOTS) strategy, which was promoted globally by the World Health Organization (WHO) starting in 1993 (23). This strategy ensured that patients completed their full treatment, effectively reducing the incidence of drug-resistant tuberculosis and helping to control the spread of the disease. The decrease in TB prevalence between 2010 and 2015 may be linked to a substantial increase in treatment coverage, which rose from 33% in 2000 to 87% in 2008. High treatment coverage is known to positively impact the reduction of TB prevalence and incidence. However, the increase in TB prevalence from 2015 to 2019 may be associated with demographic trends, such as an aging population in China (24), which presents a growing challenge for TB control. Additionally, the rise in chronic diseases, including diabetes, and the increase in rifampicin-resistant tuberculosis (RR-TB) have contributed to this upward trend (25).

The burden of TB in China showed a significant gender disparity, with a higher disease burden among males compared to females. Lifestyles such as smoking and alcohol consumption may contribute to this difference, as studies indicated that smoking is a significant risk factor for TB (26–30), and excessive alcohol consumption is also associated with an increased risk of the disease (31–34). Men typically engage in smoking and excessive drinking more than women, which could explain the higher disease burden among males. Moreover, men in China may encounter greater social and economic pressures that impact their overall health and increase their risk of TB. However, current research on this topic remains limited. Therefore, when developing prevention and control strategies, it is essential to focus on men. This includes increasing awareness of TB among males and healthcare workers (35), promoting smoking cessation (36, 37) and alcohol restriction (38), encouraging disease screening, and providing psychological support and care (39) for men.

Age had noticeable effects on the incidence and mortality rate of TB. Due to the protective effect of BCG vaccination and other factors such as low exposure risks, both the incidence and mortality rate of children under 5 years old was relatively low, and decreased with age. However, the incidence rate rose rapidly at the age of 5–15, which may be related to factors such as increase of aggregation activities of school-age children, immature immunity, and decrease of BCG protection (40). Another rapid rise stage at age of 45–65 can be due to aging since TB is an age-related disease (41). Different age groups have shown different trends of change, but the overall incidence rate and mortality rate of the older adult population were higher, suggesting that we should pay special attention to the health status of the older adult population in prevention and control work, strengthen health management, improve diagnosis and treatment level, and reduce the mortality rate. The results of the age-period-cohort model analysis also showed that the period and birth cohort have significant impacts on TB burden. The earlier the period, the higher the incidence and mortality rate, and so as the birth cohort effect. These means the epidemic situation of TB in the historical period has a far-reaching impact on the current and future disease burden. Therefore, it is necessary to fully consider the impact of historical background, e.g., the pandemic of COVID-19 has set back progress toward achieving global TB targets by several years (42, 43) and social changes including technology progress (44) on the epidemic situation of TB, so as to more accurately predict the future trend of disease burden.

Prediction of TB burden from 2022 to 2036 via the BAPC model showed that the incidence and mortality rate of TB in China would continue to decline, and the rate in males would be still higher than that in females. Despite the continuous decline, it still could not achieve the goal of “End TB by 2035” with a TB incidence of 10/100,000 people as the predicted ASIR of TB would be 21.96 (95% CI: 6.14, 37.79)/100,000 people in 2035. Meanwhile, the ASIR and ASMR for TB in 2030 would be 26.12/100,000 people (95% CI: 15.75, 36.48) and 1.13/100,000 people (95% CI: 0.45, 1.81), which showed a gap with the stop TB target in 2030. The focus of future prevention and control work should be on optimizing the relative strategies, improving diagnosis and treatment methods, and strengthening health education.

## Conclusion

This study showed that the overall TB burden in terms of ASIR, ASMR, ASPR, and ASDALYR in both sexes had decreased in the last three decades in China. However, the TB incidence predicted using the annual reduction rate via the BAPC model could not achieve the aim of “END TB by 2035,” indicating it is warranted to formulate the current strategies for preventing and controlling TB considering the factors such as gender, age, period, and birth cohort since they have important effects on the disease burden of TB. Effective strategies targeting men should focus on increasing awareness of tuberculosis among both the general population and healthcare workers. Additionally, promoting smoking cessation and alcohol moderation, enhancing disease screening and treatment access, and providing psychological support and care are crucial.

## Data Availability

Publicly available datasets were analyzed in this study. This data can be found at: https://vizhub.healthdata.org/gbd-results/.

## Footnotes

^1^ https://surveillance.cancer.gov/joinpoint/download

^2^ https://seer.cancer.gov/stdpopulations/world.who.html
