# Supplementary material for: Disease burden of tuberculosis in China from 1990 to 2021 and its prediction to 2036
Source: Front Public Health. 2025 Jan 7;12:1506266. doi: 10.3389/fpubh.2024.1506266 (PMC11747132; doi:10.3389/fpubh.2024.1506266)
Supplement: Supplementary file 1 [file Data_Sheet_1.docx]

Supplementary Material

# Supplementary Tables

**Table S1 The effect values of age, period, and cohort for the trends of burden of TB**

| **Group** | **Incidence** | | |  | **Mortality** | | |
| --- | --- | --- | --- | --- | --- | --- | --- |
|  | **RR(95%CI)** | **z** | **P value** |  | **RR(95%CI)** | **z** | **P value** |
| **Age effect** |  |  |  |  |  |  |  |
| age_0 | 0.61(0.60, 0.62) | -94.87 | <0.05 |  | 1.42(1.38, 1.46) | 26.72 | <0.05 |
| age_5 | 0.25(0.25, 0.25) | -267.90 | <0.05 |  | 0.15(0.15, 0.16) | -102.60 | <0.05 |
| age_10 | 0.41(0.40, 0.41) | -207.12 | <0.05 |  | 0.11(0.11, 0.12) | -117.96 | <0.05 |
| age_15 | 0.78(0.78, 0.79) | -68.31 | <0.05 |  | 0.17(0.17, 0.18) | -120.50 | <0.05 |
| age_20 | 0.82(0.82, 0.83) | -62.07 | <0.05 |  | 0.27(0.27, 0.28) | -111.18 | <0.05 |
| age_25 | 0.68(0.68, 0.69) | -133.49 | <0.05 |  | 0.31(0.3, 0.32) | -110.21 | <0.05 |
| age_30 | 0.69(0.68, 0.69) | -146.24 | <0.05 |  | 0.38(0.38, 0.39) | -97.35 | <0.05 |
| age_35 | 0.72(0.72, 0.73) | -136.58 | <0.05 |  | 0.46(0.45, 0.46) | -85.30 | <0.05 |
| age_40 | 0.78(0.78, 0.78) | -111.92 | <0.05 |  | 0.57(0.56, 0.58) | -66.07 | <0.05 |
| age_45 | 0.80(0.79, 0.80) | -100.17 | <0.05 |  | 0.71(0.7, 0.72) | -43.14 | <0.05 |
| age_50 | 0.92(0.92, 0.93) | -33.45 | <0.05 |  | 0.88(0.86, 0.89) | -17.59 | <0.05 |
| age_55 | 1.13(1.12, 1.14) | 48.62 | <0.05 |  | 1.14(1.13, 1.16) | 18.68 | <0.05 |
| age_60 | 1.41(1.40, 1.42) | 124.85 | <0.05 |  | 1.58(1.56, 1.60) | 66.68 | <0.05 |
| age_65 | 1.88(1.87, 1.89) | 207.20 | <0.05 |  | 2.28(2.25, 2.31) | 120.30 | <0.05 |
| age_70 | 1.78(1.77, 1.8) | 163.92 | <0.05 |  | 3.2(3.16, 3.25) | 162.13 | <0.05 |
| age_75 | 1.62(1.61, 1.63) | 117.55 | <0.05 |  | 4.13(4.07, 4.20) | 179.81 | <0.05 |
| age_80 | 1.85(1.83, 1.87) | 129.35 | <0.05 |  | 5.19(5.10, 5.28) | 182.86 | <0.05 |
| age_85 | 1.98(1.95, 2.00) | 115.04 | <0.05 |  | 5.95(5.82, 6.08) | 161.09 | <0.05 |
| age_90 | 2.01(1.98, 2.05) | 79.50 | <0.05 |  | 4.98(4.81, 5.15) | 91.20 | <0.05 |
| age_95 | 2.01(1.94, 2.09) | 36.81 | <0.05 |  | 5.31(4.93, 5.73) | 43.71 | <0.05 |
| **Period effect** |  |  |  |  |  |  |  |
| period_1992 | 1.48(1.47, 1.48) | 256.49 | <0.05 |  | 2.15(2.13, 2.16) | 208.65 | <0.05 |
| period_1997 | 1.26(1.25, 1.26) | 189.08 | <0.05 |  | 1.63(1.62, 1.64) | 144.56 | <0.05 |
| period_2002 | 1.04(1.04, 1.04) | 39.71 | <0.05 |  | 1.33(1.32, 1.34) | 87.96 | <0.05 |
| period_2007 | 0.94(0.94, 0.94) | -60.42 | <0.05 |  | 0.80(0.80, 0.81) | -57.93 | <0.05 |
| period_2012 | 0.78(0.77, 0.78) | -191.96 | <0.05 |  | 0.58(0.57, 0.58) | -125.04 | <0.05 |
| period_2017 | 0.71(0.71, 0.71) | -202.72 | <0.05 |  | 0.46(0.46, 0.47) | -148.69 | <0.05 |
| **Cohort effect** |  |  |  |  |  |  |  |
| cohort_1897 | 1.95(1.72, 2.19) | 10.84 | <0.05 |  | 3.62(3.04, 4.31) | 14.42 | <0.05 |
| cohort_1902 | 1.69(1.62, 1.76) | 23.27 | <0.05 |  | 2.95(2.76, 3.16) | 31.23 | <0.05 |
| cohort_1907 | 1.54(1.50, 1.58) | 34.68 | <0.05 |  | 2.46(2.37, 2.55) | 47.83 | <0.05 |
| cohort_1912 | 1.46(1.44, 1.49) | 40.90 | <0.05 |  | 2.15(2.09, 2.21) | 52.65 | <0.05 |
| cohort_1917 | 1.43(1.40, 1.45) | 44.67 | <0.05 |  | 2.01(1.96, 2.06) | 55.41 | <0.05 |
| cohort_1922 | 1.38(1.36, 1.39) | 44.81 | <0.05 |  | 1.9(1.86, 1.95) | 56.53 | <0.05 |
| cohort_1927 | 1.33(1.32, 1.35) | 44.28 | <0.05 |  | 1.87(1.83, 1.91) | 59.01 | <0.05 |
| cohort_1932 | 1.31(1.29, 1.32) | 44.95 | <0.05 |  | 1.8(1.76, 1.83) | 58.80 | <0.05 |
| cohort_1937 | 1.28(1.27, 1.30) | 45.41 | <0.05 |  | 1.72(1.69, 1.75) | 56.27 | <0.05 |
| cohort_1942 | 1.21(1.20, 1.22) | 38.04 | <0.05 |  | 1.53(1.51, 1.56) | 44.80 | <0.05 |
| cohort_1947 | 1.11(1.10, 1.12) | 22.34 | <0.05 |  | 1.33(1.31, 1.36) | 30.11 | <0.05 |
| cohort_1952 | 1.03(1.02, 1.04) | 6.48 | <0.05 |  | 1.19(1.17, 1.21) | 17.85 | <0.05 |
| cohort_1957 | 0.96(0.96, 0.97) | -10.18 | <0.05 |  | 1.13(1.11, 1.15) | 11.99 | <0.05 |
| cohort_1962 | 0.91(0.90, 0.91) | -29.07 | <0.05 |  | 1.01(0.99, 1.03) | 1.19 | 0.232 |
| cohort_1967 | 0.89(0.88, 0.89) | -41.78 | <0.05 |  | 0.98(0.96, 1) | -2.33 | 0.020 |
| cohort_1972 | 0.88(0.88, 0.88) | -50.12 | <0.05 |  | 0.95(0.93, 0.97) | -4.78 | <0.05 |
| cohort_1977 | 0.89(0.89, 0.90) | -49.06 | <0.05 |  | 0.91(0.89, 0.94) | -7.30 | <0.05 |
| cohort_1982 | 0.89(0.88, 0.89) | -52.21 | <0.05 |  | 0.79(0.77, 0.81) | -16.69 | <0.05 |
| cohort_1987 | 0.87(0.86, 0.87) | -66.27 | <0.05 |  | 0.68(0.66, 0.7) | -27.21 | <0.05 |
| cohort_1992 | 0.86(0.85, 0.86) | -69.05 | <0.05 |  | 0.66(0.65, 0.68) | -35.95 | <0.05 |
| cohort_1997 | 0.83(0.82, 0.83) | -62.83 | <0.05 |  | 0.54(0.53, 0.56) | -42.79 | <0.05 |
| cohort_2002 | 0.67(0.66, 0.68) | -97.76 | <0.05 |  | 0.37(0.35, 0.38) | -54.02 | <0.05 |
| cohort_2007 | 0.52(0.51, 0.53) | -116.84 | <0.05 |  | 0.25(0.24, 0.27) | -52.91 | <0.05 |
| cohort_2012 | 0.45(0.44, 0.46) | -108.88 | <0.05 |  | 0.18(0.17, 0.19) | -49.61 | <0.05 |
| cohort_2017 | 0.43(0.43, 0.44) | -87.90 | <0.05 |  | 0.11(0.1, 0.12) | -42.34 | <0.05 |
| RR: relative risk. | | | | | | | |

**Table S2 The predictive ASRs of TB from 2022 to 2036**

| **Year** | **ASIR(per 100,000 people)(95% CI)** | | |  | **ASMR(per 100,000 people) (95% CI)** | | |
| --- | --- | --- | --- | --- | --- | --- | --- |
|  | **Both sexes** | **Male** | **Female** |  | **Both sexes** | **Male** | **Female** |
| 2022 | 34.58(32.86, 36.29) | 45.16(42.68, 47.64) | 24.77(23.55, 25.99) |  | 1.71(1.61, 1.81) | 2.61(2.47, 2.75) | 0.90(0.83, 0.97) |
| 2023 | 33.38(30.87, 35.89) | 43.72(40.26, 47.18) | 23.91(22.03, 25.79) |  | 1.62(1.46, 1.79) | 2.49(2.25, 2.73) | 0.85(0.74, 0.95) |
| 2024 | 32.23(28.73, 35.72) | 42.33(37.66, 47.01) | 23.08(20.39, 25.77) |  | 1.54(1.31, 1.78) | 2.37(2.01, 2.73) | 0.80(0.65, 0.94) |
| 2025 | 31.11(26.55, 35.68) | 40.99(34.96, 47.02) | 22.28(18.72, 25.85) |  | 1.46(1.15, 1.78) | 2.26(1.78, 2.75) | 0.75(0.56, 0.94) |
| 2026 | 30.04(24.34, 35.74) | 39.68(32.22, 47.14) | 21.52(17.03, 26.01) |  | 1.39(1.00, 1.78) | 2.16(1.55, 2.77) | 0.71(0.48, 0.93) |
| 2027 | 29.00(22.14, 35.86) | 38.41(29.47, 47.35) | 20.78(15.34, 26.22) |  | 1.32(0.86, 1.79) | 2.06(1.32, 2.80) | 0.66(0.40, 0.93) |
| 2028 | 28.00(19.97, 36.03) | 37.19(26.75, 47.63) | 20.07(13.68, 26.46) |  | 1.26(0.72, 1.79) | 1.97(1.10, 2.84) | 0.62(0.32, 0.93) |
| 2029 | 27.04(17.84, 36.24) | 36.00(24.06, 47.95) | 19.39(12.03, 26.75) |  | 1.19(0.58, 1.80) | 1.88(0.88, 2.87) | 0.59(0.24, 0.93) |
| 2030 | 26.12(15.75, 36.48) | 34.86(21.41, 48.31) | 18.74(10.43, 27.05) |  | 1.13(0.45, 1.81) | 1.79(0.68, 2.91) | 0.55(0.18, 0.93) |
| 2031 | 25.23(13.71, 36.74) | 33.75(18.82, 48.69) | 18.11(8.85, 27.37) |  | 1.08(0.33, 1.82) | 1.71(0.48, 2.94) | 0.52(0.11, 0.93) |
| 2032 | 24.36(11.73, 37.00) | 32.68(16.29, 49.07) | 17.51(7.32, 27.70) |  | 1.02(0.22, 1.83) | 1.64(0.29, 2.98) | 0.49(0.05, 0.93) |
| 2033 | 23.53(9.80, 37.26) | 31.64(13.81, 49.46) | 16.93(5.83, 28.03) |  | 0.97(0.11, 1.84) | 1.56(0.12, 3.01) | 0.46(0.00, 0.92) |
| 2034 | 22.73(7.94, 37.53) | 30.63(11.41, 49.84) | 16.37(4.38, 28.36) |  | 0.92(0.01, 1.84) | 1.49(0.00, 3.04) | 0.43(0.00, 0.92) |
| 2035 | 21.96(6.14, 37.79) | 29.66(9.08, 50.23) | 15.83(2.98, 28.68) |  | 0.88(0.00, 1.85) | 1.43(0.00, 3.07) | 0.41(0.00, 0.91) |
| 2036 | 21.22(4.40, 38.03) | 28.71(6.83, 50.59) | 15.31(1.63, 29.00) |  | 0.83(0.00, 1.85) | 1.36(0.00, 3.09) | 0.38(0.00, 0.91) |
| When predicting ASRs for a given year, if the lower limit of the 95% confidence interval is below 0, it is set to 0.  ASR: Age-standardized rate,ASIR: Age-standardized incidence rate, ASMR: Age-standardized mortality rate, CI: confidence interval, TB: tuberculosis. | | | | | | | |
|  | |  |  |  |  |  |  |
